# Vascular encasement image defined risk factors independently predict surgical complications in neuroblastoma

**DOI:** 10.1111/ans.19420

**Published:** 2025-01-30

**Authors:** Rachael Stokes, Aidan Bannon, Bonnie Leung, Jasmin Alloo, David Davies‐Payne, Mark Winstanley, Andrew Wood, Stephen Evans, James Hamill

**Affiliations:** ^1^ Department of Paediatric Surgery Starship Children's Hospital Auckland New Zealand; ^2^ Department of Radiology Starship Children's Hospital Auckland New Zealand; ^3^ Department of Oncology Starship Children's Hospital Auckland New Zealand; ^4^ Faculty of Medical and Health Sciences, Molecular Medicine and Pathology University of Auckland Auckland New Zealand; ^5^ Faculty of Medical and Health Sciences, Paediatrics, Child and Youth Health University of Auckland Auckland New Zealand

**Keywords:** image defined risk factors, neuroblastoma, postoperative complications, surgical oncology

## Abstract

**Background:**

Specific image defined risk factors (IDRF) immediately prior to surgery may be more relevant to paediatric oncology surgeons than pre‐neoadjuvant IDRFs at diagnosis. The aim of this study was to determine IDRF subtypes that independently predict postoperative complications.

**Methods:**

We searched the New Zealand Children's Cancer Registry for all cases of neuroblastoma treated at a single paediatric oncology centre between January 2007 and February 2021 and determined the IDRF status on pre‐operative imaging at diagnosis and after neoadjuvant therapy. Surgical complications (Clavien–Dindo grade) were correlated with total number of IDRFs (pre‐ and post‐chemotherapy) and three subsets: vascular encasement (VE), invasive (I), and extensive (E).

**Results:**

Of 101 patients, 73 underwent surgical resection, and 32 (44%) had a surgical complication. Of the 54 IDRF‐positive tumours, all were treated by neoadjuvant therapy and in 17, all IDRFs resolved. Complications correlated with the number of post‐neoadjuvant therapy VE‐IDRFs at OR 1.2 (95% CI 1.0–1.4, *P* = 0.02) and extensive IDRFs at OR 1.7 (95% CI 1.1–1.9, *P* = 0.02). Pre‐neoadjuvant IDRF status was not independently associated with complications when controlling for post‐neoadjuvant IDRF status. The total number of VE‐IDRF reduced from 181 pre‐neoadjuvant therapy to 86 post, with tumour encasing the aorta and/or vena cava being the most common.

**Conclusions:**

The vascular encasement and extensive subtypes of IDRF may be more useful prognostic indicators of surgical complications than the total number of IDRFs. This may have implications for reporting IDRF status on preoperative imaging and surgical planning but needs validation in larger cohort studies.

## Graphical abstract

This single centre retrospective study supports a correlation between vascular encasement image defined risk factors post neoadjuvant chemotherapy and surgical complications, in patients with neuroblastoma. Reporting IDRF subtypes may facilitate surgical planning and minimize post‐operative complications.

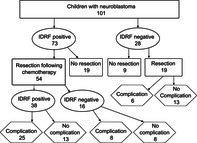

## Introduction

Image defined risk factors (IDRF) play a vital role in neuroblastoma staging and risk classification. The IDRF system evolved from ‘surgical risk factors’ used by the European International Society of Paediatric Oncology Neuroblastoma Group. Cecchetto *et al*. showed that surgical risk factors increased the rate of postoperative complications and decreased the chance of complete surgical resection.^1^ Surgical risk factors were renamed IDRF and incorporated into the International Neuroblastoma Risk Group Staging System (INRGSS) which consists of four stages: L1, L2, M, and MS, where stage L1 represents localized tumour with no IDRF present and Stage L2 represents localized neuroblastoma with one or more IDRF.^2^ The INRGSS in turn contributes to the International Neuroblastoma Risk Group (INRG) staging system in which the patient's age, tumour stage, histology, and genomic biomarkers (e.g., *MYCN* status) categorize patients into four risk groups: very low‐risk, low‐risk, intermediate‐risk, and high‐risk. This was revised in 2021 to also incorporate single chromosome aberration status.^3^ Therefore, IDRF contributes to the oncological risk classification of children with neuroblastoma.

IDRF status influences surgical planning; for example, tumours with no IDRF can be resected up front while IDRF‐positive tumours receive neoadjuvant therapy followed by surgery. Completeness of resection in high‐risk tumours is advised because complete or near‐complete surgical resection may improve survival.^4^, ^5^ Complete resection is inversely related to the number of IDRFs present after neoadjuvant therapy.^6^, ^7^, ^8^ IDRF status also influences the risk of complications after surgery.^8^, ^9^, ^10^, ^11^ Of emerging interest is how specific types of IDRF, not just the presence or absence of an IDRF, influence surgical complications. Temple *et al*. showed that vascular encasement IDRFs, where tumour surrounds a major blood vessel by more than 50%, correlate with postoperative complications^12^; however, this was not confirmed by van Heerden *et al*. who found instead that postoperative complications were significantly more likely with organ invasion IDRFs and tumour in two body compartments (the ‘extensive’ IDRF subtype).^13^ Therefore, the significance of specific types of IDRF is not entirely clear; as noted by Cecchetto *et al*., more work is needed on how IDRF type influences the risk of particular complications.^1^

The purpose of this study was to correlate specific types of IDRF with complications following neuroblastoma resection. A secondary aim was to document the change in IDRF status following neoadjuvant therapy and determine whether post‐chemotherapy IDRF status was more predictive of postoperative complications than pre‐chemotherapy IDRF status.

## Methods

This study was approved by the Auckland District Health Board Research Office (A+8215) and registered on the Open Science Framework (osf.io/3x6r9/). We searched the New Zealand Children's Cancer Registry for all cases of neuroblastoma treated at our paediatric oncology centre between January 2007 and February 2021. Our centre is a tertiary referral children's hospital and one of two paediatric oncology centres in New Zealand. Over the study period, two paediatric surgeons performed the majority of neuroblastoma resections.

Data collected included patient demographics, tumour characteristics, IDRF status, and surgical details. Two consultant paediatric radiologists (BL and DDP) performed radiological assessment of IDRF status by reviewing preoperative imaging and, where relevant, imaging following neoadjuvant chemotherapy. These radiologists classified IDRF using published guidelines^14^, ^15^ as per Monclair *et al*., using radiological standard definitions; ‘vascular’ encasement meaning more than 50% of the vessel circumference is in contact with the tumour, ‘invasive’ (infiltration) referring to the involvement of vital structures other than vessels, and ‘extensive’ involving two body compartments. Eight IDRFs were classified as ‘vascular’, three as ‘invasive’, and one as ‘extensive’ as shown in Table S2.

Complications were recorded including those documented in the operation note, discharge summary, or clinic letters and were classified according to standard Clavien–Dindo (CD) grading.^16^

For statistical analysis, the grade of complication was correlated with the number of IDRFs. The number of high‐grade complications was small; therefore, the complications were converted into a binomial variable comparing CD 0–I in one group to CD II–IV in the ‘complication’ group. The distribution of continuous variables was tested for normality using the Shapiro–Wilk test and density plots. Analysis was performed using the Mann–Whitney *U* test, Fisher's exact test, or generalized additive models, as appropriate, to account for the non‐parametric distribution of the data. The number of IDRFs pre‐ and post‐chemotherapy, patient's age at diagnosis, histology (favourable or unfavourable), *MYCN* amplification, loss of *CHD5*, and loss of *ATM* were examined in the models. Risk Group was not included in the model because IDRF status, age, and histology (which contribute to Risk Group) were already included. Models were run for the IDRF subgroups vascular encasement (‘vascular’), organ invasion (‘invasive’), extension within two body compartments (‘extensive’), and ‘other’, that is, not vascular, invasive, or extensive (see Table S2 for a list of IDRFs and types). *P*‐values were obtained by comparison with a null model using analysis of variance. Effect sizes (log‐odds) were exponentiated to odds ratios (OR) and reported with 95% confidence interval (95% CI). Statistical analysis was performed in R^17^ using the packages tidyverse^18^ and mgcv.^19^

## Results

### Patient and tumour characteristics

The registry included 101 children with neuroblastoma between 2007 and 2021, of whom 73 underwent surgical resection of their tumour. Of the 28 patients who did not have surgery, six had surveillance for Stage L1, two had surveillance for Stage MS, 18 had Stage M, and two were lost to follow up (relocated to Perth and Tonga). Of the 26 patients with data available, 12 were deceased, all of whom had metastatic disease. The median age at diagnosis was 23 months (range 0–175 months); 56 were 18 months of age or older. Patient and tumour characteristics by the presence or absence of IDRF are shown in Table S1, and patient flow chart in Figure S1.

### Surgery

In total, 73 patients had a surgical procedure for their tumour. Completeness of resection, as estimated by the surgeon, was 100% in 31, 90%– 99% in 31, and debulking or biopsy only in the remainder. Surgical complications occurred in 39: 5 Grade I; 19 Grade II; 13 Grade III; 2 Grade IV; no Grade V (mortality) surgical complications.

### Image defined risk factors

At diagnosis, 73 tumours had one or more IDRF with a median number of IDRFs of 3 (IQR 2–6, range 1–9) of which 50 had a ‘vascular’ IDRF, 36 had an ‘invasive’ IDRF, and 18 had an ‘extensive’ IDRF. Fifty‐four tumours had IDRF status re‐evaluated after neoadjuvant therapy of which 38 had one or more IDRF with a median number of IDRFs of 2 (IQR 1–3, range 1–6); 16 tumours lost all their IDRFs. Eighteen tumours had a ‘vascular’ IDRF, 23 had an ‘invasive’ IDRF, and 8 had an ‘extensive’ IDRF as shown in Table S2.

The number of IDRFs was significantly associated with unfavourable histology (*P* = 0.03) but not with *MYCN* amplification, loss of *CHD5*, or loss of *ATM*. The change in the number of IDRFs after chemotherapy was significantly associated with the number of IDRFs before chemotherapy (*P* < 0.001) but was not independently associated with unfavourable histology, *MYCN* amplification, loss of *CHD5*, or loss of *ATM*.

### Surgical complications

**Fig. 1:**
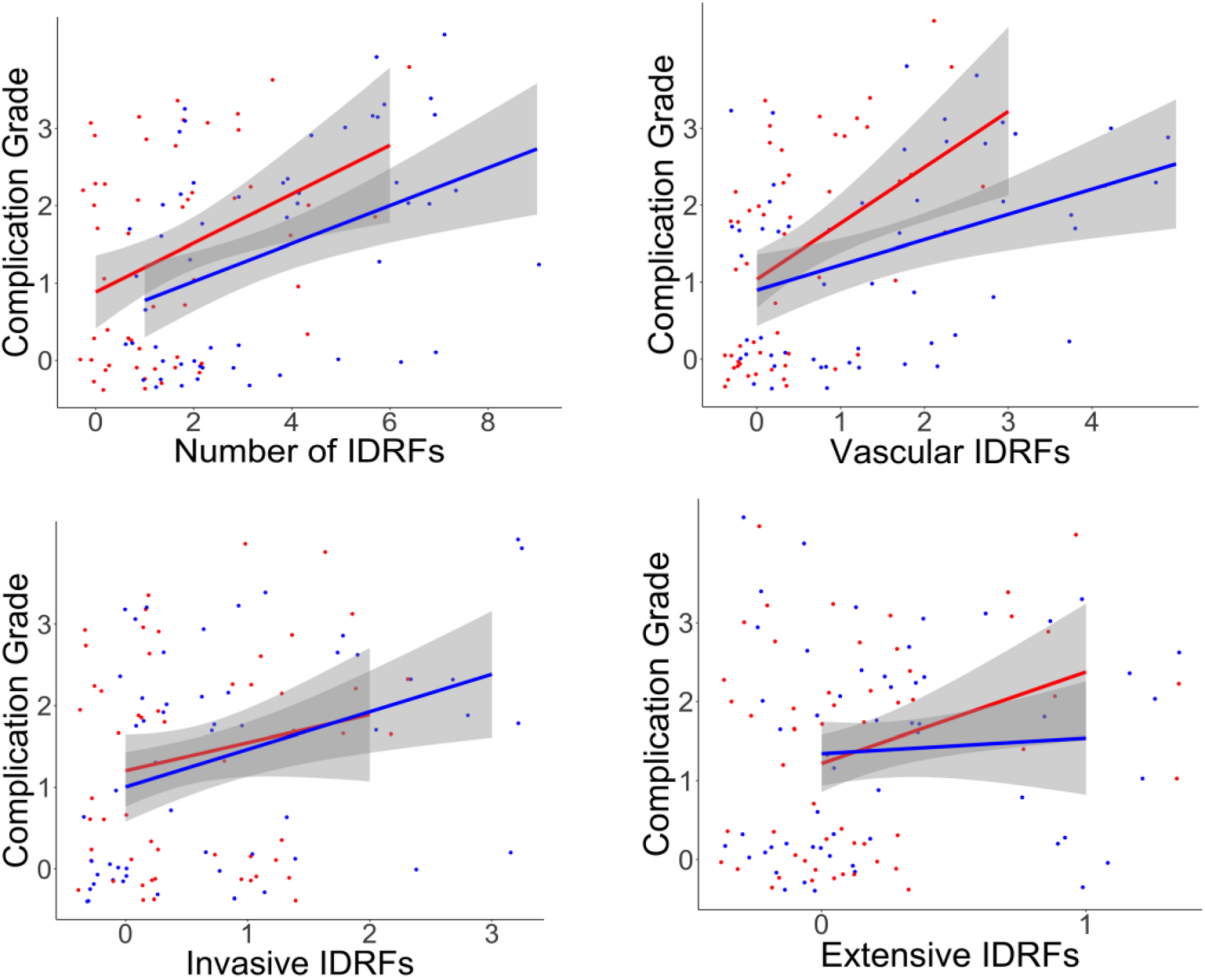
Image defined risk factors (IDRFs) before and after neoadjuvant therapy by Clavien‐Dindo grade of complication with regression lines and 95% confidence limits (grey shade). Blue (lower) line, IDRFs at diagnosis. Red (upper) line, IDRF status following neoadjuvant therapy. Top left, total number of IDRFs. Top right, vascular encasement IDRFs. Bottom left, invasive IDRFs. Bottom right, IDRFs other than those of the vascular encasement and invasive (extensive subtype).

On univariate analysis, surgical complications were significantly associated with the number of IDRFs, both before and after chemotherapy, as shown in Tables 1, S3, and Figure 1. On initial imaging, vascular and invasive IDRF subtypes were significantly associated with complications, while on imaging following neoadjuvant chemotherapy, the vascular IDRF subtype was significantly associated with complications.

**Table 1:** Univariate analysis of complications versus no complications in those who underwent surgical resection, categorical variables.

	No complication (*n* = 39)	Complication (*n* = 34)	*P‐*value	OR [95% CI]
Age				
<18 months	22	9	0.02*	3.5 [1.2, 11]
≥18 months	17	25		
Risk group				
Low risk	10	6	0.02*	3.2 [1.1, 9.4]
Intermediate risk	16	7		
High risk	13	21		
Histology				
Favourable	23	11	0.03*	3.3 [1.1, 10]
Unfavourable	13	21		
MYCN status				
Not amplified	31	30	0.3	0.4 [0.1, 2.2]
Amplified	7	3		
CHD5 status				
No LOH	21	23	0.6	0.7 [0.2. 2.2]
LOH	12	9		
ATM status				
No LOH	17	13	0.8	1.2 [0.3, 4.2]
LOH	11	10		
Resection extent				
100%	21	10	0.03*	2.9 [1.0, 8.9]
90%–99%	13	18		
<90%	4	6		
IDRF at diagnosis				
Any IDRF	26	28	0.003*	1.07 [1.02, 1.12]
Vascular subtype	16	19	0.003*	1.12 [1.04, 1.21]
Invasive subtypes	9	18	0.002*	1.20 [1.07, 1.35]
Extensive subtype	6	7	0.8	1.42 [0.36, 5.78]
Other subtypes	15	13	0.9	1.00 [0.89, 1.11]
IDRF post‐chemotherapy				
Any IDRF	17	21	0.02*	1.10 [1.01, 1.20]
Vascular subtypes	4	14	0.005*	1.27 [1.07, 1.50]
Invasive subtypes	10	13	0.1	1.17 [0.96, 1.42]
Extensive subtype	2	6	0.2	3.21 [0.50, 35.7]
Other subtypes	9	8	0.7	0.97 [0.79, 1.17]

*Note*: ‘Complication’ is defined as Clavien‐Dindo^16^ grade II or above. Statistical analysis by Fisher's exact test. Risk Group compares high risk to intermediate/low risk groups. Resection extent compares 100% to <100% resection groups. Seventy three patients had a surgical procedure of which 55 underwent their procedure following neoadjuvant therapy.

* Statistically ‘significant’ results.

On multivariate analysis of IDRFs assessed on imaging at diagnosis, controlling for age and histology, complications were significantly associated with vascular (OR 1.1, *P* = 0.02) and invasive (OR 1.13, *P* = 0.05) subtypes. On imaging following neoadjuvant chemotherapy, complications were significantly associated with vascular (OR 1.2, *P* = 0.02) and extensive (OR 1.7, *P* = 0.02) subtypes, as shown in Table 2. When both pre‐ and post‐chemotherapy IDRFs were included in the model, only post‐chemotherapy IDRFs trended towards independent association with complications (*P* = 0.05) while pre‐chemotherapy IDRFs were not independently associated with complications (*P* = 0.4).

**Table 2:** Multivariate analysis of the risk of a complication showing odds estimates (obtained by exponentiating the log odds outputs from the models), 95% confidence intervals (obtained by calculating the standard error), and *P*‐values obtained using anova by comparing models with the null model that does not have the IDRF parameter).

	Estimate (odds)	*P‐*value
Pre‐chemotherapy		
All	1.06 [1.01, 1.12]	0.01*
Vascular	1.10 [1.01, 1.20]	0.02*
Invasive	1.13 [0.99, 1.30]	0.05*
Extensive	1.02 [0.73, 1.41]	0.9
Others	1.05 [0.92, 1.18]	0.4
Post‐chemotherapy		
All	1.12 [1.02, 1.23]	0.01*
Vascular	1.20 [1.00, 1.44]	0.02*
Invasive	1.10 [0.89, 1.35]	0.4
Extensive	1.66 [1.09, 1.94]	0.02*
Others	1.11 [0.88, 1.41]	0.3

*Note*: Pre‐chemotherapy IDRFs were based on imaging at presentation (*n* = 73); post‐chemotherapy IDRFs were based on imaging following neoadjuvant chemotherapy (*n* = 54).

* Statistically ‘significant’ results.

## Discussion

This study indicates that IDRF status following neoadjuvant therapy predicts postoperative complications independent of the pre‐chemotherapy IDRF status. Additionally, some subtypes of IDRFs are more predictive of complications than others. Irtan *et al*. first showed that IDRFs change considerably after chemotherapy,^6^ subsequently confirmed by others.^20^, ^21^ IDRF status at diagnosis contributes to neuroblastoma staging; L2 (or M if metastases) generally receive neoadjuvant therapy followed by further imaging and, if feasible, surgical resection. This study shows that only the post‐chemotherapy IDRF status independently predicts surgical complications.

IDRF status is known to correlate with surgical complications,^9^, ^22^ resectability,^6^ and survival.^23^ Of emerging importance is the association of postoperative complications with specific IDRF groups, for example, those of the vascular encasement type,^12^ those of the organ invasive type,^13^ or tumour in multiple body compartments.^13^ The latter subtype (‘extensive’) have been previously reported to be associated with an increased risk of postoperative complications,^13^ as shown in the present study. Interestingly, in contrast to our study, van Heerden *et al*. found that organ invasion was significantly correlated with complications but vascular encasement was not, indicating the limitations of small retrospective series and the need for large multicentre studies on IDRF subtypes. To our knowledge, ours is the first study to take a multivariate approach and, therefore, adds weight to previous work in this area.

One value of the IDRF system is that surgical challenges are translated for the rest of the oncology team and incorporated into staging and risk grouping. A more nuanced understanding of specific IDRF could facilitate collaboration between the paediatric surgeon, radiologist, and oncologist. Our findings confirm that not all IDRFs are equal^6^, ^8^ by showing the independent association of vascular encasement IDRFs with complications.^12^, ^24^ IDRF status not only correlates with general complications such as haemorrhage but also with specific complications such as chyle leak. This suggests that in planning neuroblastoma resection, radiologists and surgeons should work together to highlight particular IDRFs that might increase the risk of particular complications and plan steps to mitigate this risk. Radiological reporting of neuroblastomas could highlight specific IDRFs using a standardized neuroblastoma reporting format along the lines of the recently published International Neuroblastoma Surgical Reporting Form.^25^ It would be helpful for less experienced paediatric oncology surgeons to be wary of specific complications associated with certain IDRFs and avoid these.

Multiple case series have recently looked at patient‐specific 3D anatomical models, and other augmented reality visualization techniques, to improve paediatric surgeon's understanding of anatomical relationships and pre‐operative planning in complex oncological cases.^26^, ^27^, ^28^ Fitski *et al*. and Simons *et al*. from the Netherlands showed promising results in nephron‐sparing surgery for Wilms Tumour and the use of multi‐modal 3D heatmaps for neuroblastoma respectively.^26^, ^27^ Major limitations of these techniques include cost (US$400–€3000) and time (hours to days), and they are not yet widely available, nor have they been validated in large prospective studies.^29^ However, once validated, a better understanding of which IDRF's are significant, in terms of surgical complications, may help to identify which patients would benefit from these techniques, despite the cost and time.

## Limitations

This study was limited by its relatively small size. The association of non‐vascular IDRFs and invasive IDRFs with complications may have reached statistical significance with a larger sample size. It was a single‐centre experience. We determined complications retrospectively. Our complication rate was higher than some previous series (9%–28%)^1^, ^9^ but not as high as others (up to 84%)^11^ suggesting that overall complication rates might depend on the definition of a complication used in the particular series. In the largest published series to date, 30% had surgical risk factors, and complications were reported simply as nonfatal or fatal.^1^ A strength of our complication analysis was the use of the Clavien–Dindo grading system. Our retrospective dataset did not lend itself to a full analysis of the completeness of resection as an outcome because postoperative imaging was not routinely performed in all cases; completeness of resection in relation to IDRF status has been well described by others.^5^, ^6^ Data were incomplete for biological markers including loss of *CDH5* on chromosome 1p and loss of *ATM* on chromosome 11q because risk stratification criteria changed during the study period.

## Conclusions

Specific IDRFs are of more importance to the surgeon than the presence or absence of any IDRF. Larger cohort studies are needed to elicit the true correlation between complications and specific subtypes of IDRFs, and the role that standardized reporting of IDRF status could play in improving surgical outcomes. Close collaboration between oncologist, radiologist, and surgeon is recommended in preoperative planning of neuroblastoma resection in the presence of IDRFs.

## Author contributions

**Rachael Stokes:** Investigation; writing – original draft; writing – review and editing. **Aidan Bannon:** Investigation. **Bonnie Leung:** Investigation. **Jasmin Alloo:** Investigation. **David Davies‐Payne:** Investigation. **Mark Winstanley:** Writing – review and editing. **Andrew Wood:** Writing – review and editing. **Stephen Evans:** Writing – review and editing. **James Hamill:** Conceptualization; data curation; formal analysis; supervision; writing – original draft; writing – review and editing.

## Funding information

No funding was received for this study.

## Conflict of interest

None declared.

## Supporting information


**Data S1.** Supporting Information.

**Figure S1.** Flow chart of patients by image defined risk factor (IDRF) status, surgery, and complications.

**Table S1.** Baseline characteristics and univariate analysis by image defined risk factor (IDRF) status.

**Table S2.** List of Image Defined Risk Factors (IDRF) with vascular encasement and organ invasion subtypes. Figures represent the number of IDRFs at diagnosis and after neoadjuvant therapy (pre → post); *n* = 54.

**Table S3.** Complication rates related to the presence or absence of any IDRF, or IDRF subtype, based on imaging at presentation (‘pre‐chemotherapy’, *n* = 73) and imaging following neoadjuvant chemotherapy (‘post‐chemotherapy’, *n* = 54) (univariate analysis, Fisher's exact test).
