# Supplementary material for: Vascular encasement image defined risk factors independently predict surgical complications in neuroblastoma
Source: ANZ J Surg. 2025 Jan 30;95(6):1147–52. doi: 10.1111/ans.19420 (PMC12227851; doi:10.1111/ans.19420)
Supplement: Supplementary file 1 — Data S1. Supporting Information. [file ANS-95-1147-s005.docx]

**Supporting Information: Description of Complications**

Amongst the complications were four chyle leaks (one thoracic and three abdominal), three haemorrhages secondary to retroperitoneal vessel injury (right gonadal artery, left common iliac vein, left renal vein), two small bowel obstructions requiring adhesiolysis, obstructive jaundice (requiring cholecystostomy tube placement), two nerve injuries (right vocal cord palsy and left phrenic nerve injury), one case of bilateral pneumothoraces, and one case of transection of the common iliac vein.

All four children with chyle leaks received five cycles of neoadjuvant therapy. The three patients with abdominal chyle leaks all had encasement of the aorta or inferior vena cava (IDRF number 16) and encasement of the origin of the coeliac axis or origin of SMA (IDRF number 14). Two of these patients underwent relook laparotomy after the failure of medical management by percutaneous drainage and intravenous nutrition. In both children, surgeons identified that the source of the chyle leak was at the origin of the celiac axis and successfully managed the leak by suture ligation. The third patient’s chyle leak responded to medical management without requiring surgical repair. The chylothorax patient was IDRF-positive for infiltration of costovertebral junction T9 – T12 (IDRF number 10) and ipsilateral extension of the tumour within two body compartments (IDRF number 1).
