# Supplementary material for: Vascular encasement image defined risk factors independently predict surgical complications in neuroblastoma
Source: ANZ J Surg. 2025 Jan 30;95(6):1147–52. doi: 10.1111/ans.19420 (PMC12227851; doi:10.1111/ans.19420)

**Figure S1**

Flow chart of patients by image defined risk factor (IDRF) status, surgery, and complications.


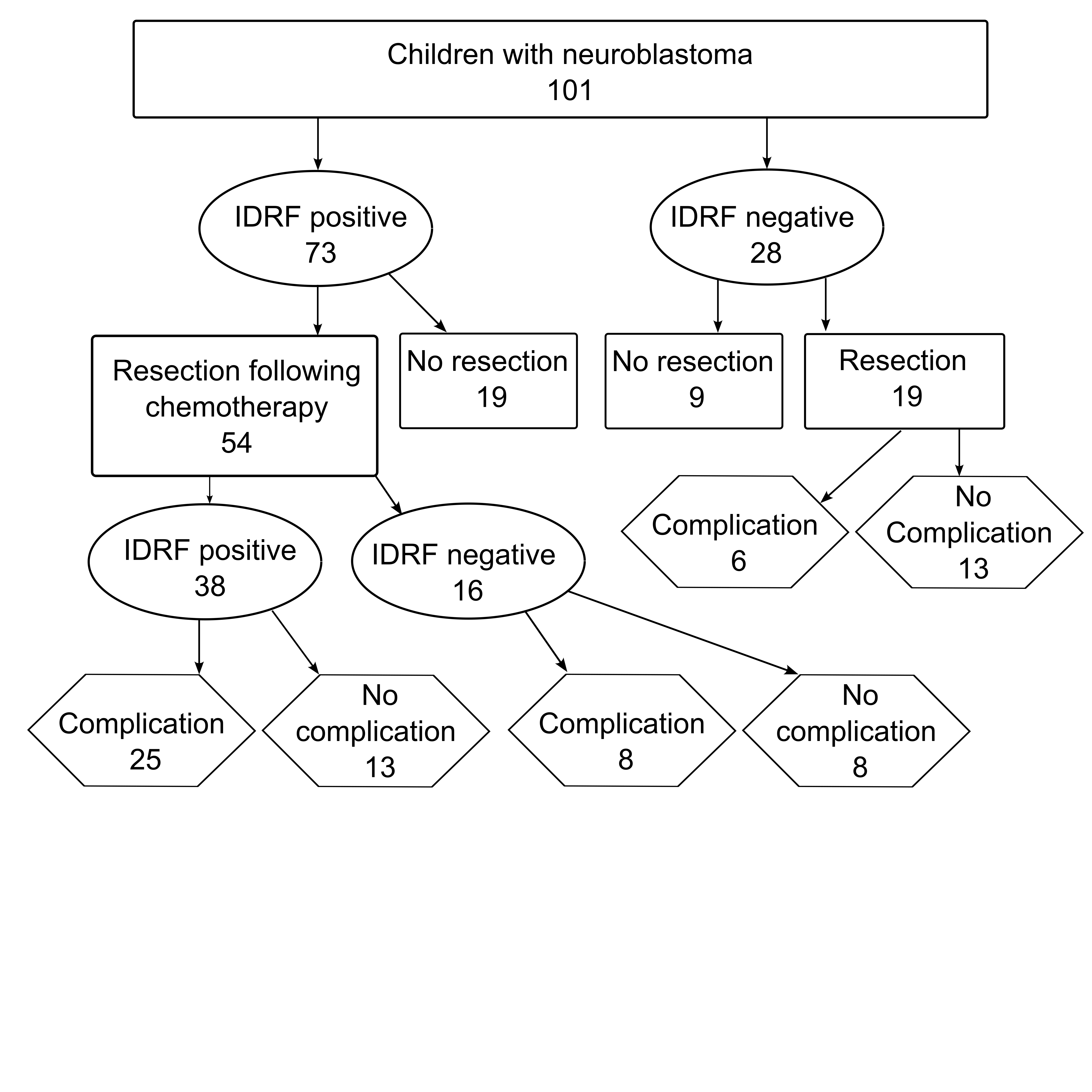

Supplement: Supplementary file 2 — Figure S1. Flow chart of patients by image defined risk factor (IDRF) status, surgery, and complications. [file ANS-95-1147-s003.docx]
