# Supplementary material for: Vascular encasement image defined risk factors independently predict surgical complications in neuroblastoma
Source: ANZ J Surg. 2025 Jan 30;95(6):1147–52. doi: 10.1111/ans.19420 (PMC12227851; doi:10.1111/ans.19420)
Supplement: Supplementary file 3 — Table S1. Baseline characteristics and univariate analysis by image defined risk factor (IDRF) status. [file ANS-95-1147-s002.docx]

**Table S1.** Baseline characteristics and univariate analysis by image defined risk factor (IDRF) status.

|  | All patients* n = 101 | IDRF-negative  n = 28 | IDRF-positive  n = 73 | *p* value |
| --- | --- | --- | --- | --- |
| Age |  |  |  |  |
| Median months [IQR]) | 23 [9, 42] | 9.5 [0, 30] | 26 [12, 43] | 0.001 |
| Gender |  |  |  |  |
| Female  Male | 49 52 | 12 16 | 37 36 | 0.5 |
| Ethnicity |  |  |  |  |
| Māori  Pacific  European  Other | 21 10 61 9 | 3  3  18  4 | 18  7  43  5 | 0.3 |
| Site |  |  |  |  |
| Abdominal/Pelvic  Thoracic  Neck | 75  21  5 | 23  3  2 | 52  18  3 | 0.3 |
| Side |  |  |  |  |
| Left  Right  Midline | 47  39  14 | 12  13  2 | 35  26  12 | 0.4 |
| INRG Stage |  |  |  |  |
| L1  L2  M  MS | 20 33 43 5 | 20  0  5  3 | 0  33  38  2 | 0.003 |
| INRG Risk Group |  |  |  |  |
| Low Risk  Intermediate Risk  High Risk | 20  31  48 | 16  6  6 | 4  25  42 | <0.0001 |
| Histology |  |  |  |  |
| Unfavourable  Favourable | 48  44 | 9  17 | 39  27 | 0.04 |
| *MYCN* amplification |  |  |  |  |
| Positive  Negative | 15  81 | 6  19 | 9  62 | 0.2 |
| Loss of *CHD5* |  |  |  |  |
| Yes  No | 31  57 | 6  17 | 25  40 | 0.3 |
| Loss of *ATM* |  |  |  |  |
| Yes  No | 23  36 | 3  15 | 20  21 | 0.02 |
| Surgical resection |  |  |  |  |
| No  Yes | 73  28 | 19  9 | 54  19 | 0.6 |
| Resection extent |  |  |  |  |
| 100%  90 – 99%  <90% | 31  31  10 | 15  3  1 | 16  28  9 | 0.001 |

Age refers to age at diagnosis. INRGSS, International Neuroblastoma Risk Group Staging System. IDRF, Image Defined Risk Factors.*Data in the all patients column may not total 101 due to missing data points: side not available in 1; INRG Risk Group not available in 2; histology not available in 9; *MYCN* data not available in 5; *CHD5* data not available in 13, *ATM* data not available 42, resection not performed in 29.
