# Supplementary material for: Vascular encasement image defined risk factors independently predict surgical complications in neuroblastoma
Source: ANZ J Surg. 2025 Jan 30;95(6):1147–52. doi: 10.1111/ans.19420 (PMC12227851; doi:10.1111/ans.19420)
Supplement: Supplementary file 4 — Table S2. List of Image Defined Risk Factors (IDRF) with vascular encasement and organ invasion subtypes. Figures represent the number of IDRFs at diagnosis and after neoadjuvant therapy (pre → post); n = 54. [file ANS-95-1147-s001.docx]

**Table S2**

List of Image Defined Risk Factors (IDRF) with vascular encasement and organ invasion subtypes. Figures represent the number of IDRFs at diagnosis and after neoadjuvant therapy (pre → post); n = 54.

| # | Location | Image Defined Risk Factor | Pre → Post | Vascular | Invasive |
| --- | --- | --- | --- | --- | --- |
| 1 | Ipsilateral tumour extension within 2 body compartments (‘extensive’) | Neck-chest, chest-abdomen, abdomen-pelvis | 13 → 8 |  |  |
| 2 | Neck | Tumour encasing carotid and/or vertebral artery and/or internal jugular vein | 3 → 1 | ✓ |  |
| 3 |  | Tumour extending to base of skull | 4 → 1 |  |  |
| 4 |  | Tumour compressing the trachea | 1 → 0 |  |  |
| 5 | Cervico-thoracic junction | Tumour encasing brachial plexus roots | 5 → 1 |  |  |
| 6 |  | Tumour encasing subclavian vessels and/or vertebral and/or carotid artery | 6 → 2 | ✓ |  |
| 7 |  | Tumour compressing the trachea | 4 → 1 |  |  |
| 8 | Thorax | Tumour encasing the aorta and/or major branches | 4 → 3 | ✓ |  |
| 9 |  | Tumour compressing the trachea and/or principal bronchi | 12 → 4 |  |  |
| 10 |  | Lower mediastinal tumour, infiltrating the costo-vertebral junction between T9 and T12 | 13 → 7 |  |  |
| 11 | Thoraco-abdominal | Tumour encasing the aorta and/or vena cava | 18→ 2 | ✓ |  |
| 12 | Abdomen/pelvis | Tumour infiltrating the porta hepatis and/or the hepatoduodenal ligament | 10 → 0 |  | ✓ |
| 13 |  | Tumour encasing branches of the superior mesenteric artery at the mesenteric root | 10 → 0 | ✓ |  |
| 14 |  | Tumour encasing the origin of the celiac axis, and/or of the superior mesenteric artery | 16 → 4 | ✓ |  |
| 15 |  | Tumour invading one or both renal pedicles | 18 → 21 |  | ✓ |
| 16 |  | Tumour encasing the aorta and/or vena cava | 28 → 11 | ✓ |  |
| 17 |  | Tumour encasing the iliac vessels | 6 → 3 | ✓ |  |
| 18 |  | Pelvic tumour crossing the sciatic notch | 0 → 1 |  |  |
| 19 | Intraspinal tumour extension whatever the location provided that: | More than one third of the spinal canal in the axial plane is invaded and/ or the perimedullary leptomeningeal spaces are not visible and/or the spinal cord signal is abnormal | 12 → 8 |  |  |
| 20 | Infiltration of adjacent organs/structures | Pericardium, diaphragm, kidney, liver, duodeno-pancreatic block, and mesentery | 17 → 8 |  | ✓ |
|  | Total |  | 189→ 86 | 81 → 26 | 45 → 29 |

Source: Monclair T, Brodeur GM, Ambros PF, Brisse HJ, Cecchetto G, Holmes K, et al. The International Neuroblastoma Risk Group (INRG) Staging System: An INRG Task Force Report. J Clin Oncol 2009;27:298–303. https://doi.org/10.1200/JCO.2008.16.6876.
