# Supplementary material for: Vascular encasement image defined risk factors independently predict surgical complications in neuroblastoma
Source: ANZ J Surg. 2025 Jan 30;95(6):1147–52. doi: 10.1111/ans.19420 (PMC12227851; doi:10.1111/ans.19420)
Supplement: Supplementary file 5 — Table S3. Complication rates related to the presence or absence of any IDRF, or IDRF subtype, based on imaging at presentation (‘pre‐chemotherapy’, n = 73) and imaging following neoadjuvant chemotherapy (‘post‐chemotherapy’, n = 54) (univariate analysis, Fisher's exact test). [file ANS-95-1147-s004.docx]

**Table S3.** Complication rates related to the presence or absence of any IDRF, or IDRF subtype, based on imaging at presentation (‘pre-chemotherapy’, n = 73) and imaging following neoadjuvant chemotherapy (‘post-chemotherapy’, n = 54) (univariate analysis, Fisher’s exact test).

|  | IDRF positive | IDRF negative | *p* value |
| --- | --- | --- | --- |
| Pre-chemotherapy |  |  |  |
| Any IDRF | 28/54 (52%) | 6/19 (32%) | 0.2 |
| Vascular | 19/35 (54%) | 15/38 (39%) | 0.2 |
| Invasive | 18/27 (67%) | 16/46 (35%) | 0.01 |
| Extensive | 7/13 (54%) | 37/60 (62%) | 0.8 |
| Post-chemotherapy |  |  |  |
| Any IDRF | 21/38 (55%) | 7/16 (44%) | 0.6 |
| Vascular | 14/18 (78%) | 14/36 (39%) | 0.01 |
| Invasive | 13/23 (57%) | 15/31 (48%) | 0.6 |
| Extensive | 6/8 (75%) | 22/46 (48%) | 0.3 |
